# Aging, Physical Activity, and Disease Prevention 2012

**DOI:** 10.1155/2012/373294

**Published:** 2012-12-09

**Authors:** Iris Reuter

**Affiliations:** Department of Neurology, Justus-Liebig University Giessen, 35392 Giessen, Germany

Sociodemographic changes have led to an increase of aging in the society [1]. The biological and physiological changes of aging are primarily associated with a decline in muscle mass ranging from 1% to 2% per year past the age of 50, strength, endurance, and the inability to maintain balance [2]. Furthermore, the prevalence and incidence of cardiovascular diseases, diabetes mellitus, osteoarthritis, and neurodegenerative diseases rise with age resulting in a slowing of movements, imbalance, immobility, falls, and disability. Recent studies revealed an increase of disability among the older population [3, 4]. Age older than 84 years, lower education levels, obesity, comorbid conditions, not practicing physical activity, and sleeping more than 8 hours per day have been associated with higher disability [4]. Some studies have shown that elderly women are less active as well. Self-perceived health is worse in subjects with a greater number of comorbid conditions and disability and is considered a barrier for participation in exercise programmes [5]. Age of 80 years and beyond, more than 2 comorbid chronic conditions, and obesity have been shown to be associated with a lower likelihood of practicing leisure time physical activity [6]. Similarly, older people (age ≥ 80 years), those taking a greater number of medications for chronic conditions, obese, and with worse self-perceived health status tended to have a relatively lower physical fitness [5].

Besides health conditions, other factors might also affect physical activity. Some studies have shown that people who were physically active throughout their life keep the habit of exercising in old age. However, the time potentially available for leisure time physical activity depends on the amount of time required for paid employment, family, and daily mobility requirements. The time budget seems to be the most limited for middle-aged adults with job and family. Thus, many middle-aged adults give up leisure time sports and do not start participating in sports again [7, 8]. A sedentary life style is an independent risk factor for cardiovascular diseases, diabetes mellitus and musculoscelettal disorders [9]. 

There is a growing body of literature showing that regular exercise benefits well-being and health condition and leads to an increasing quality of life. Physical activity (PA) has been considered one key element for determining health status [3, 10]. Furthermore, physical activity improves mood and cognitive function [11]. Older adults who are physically active are c. 21%  (*P* ≤ .05) less likely than their counterparts to be diagnosed with dementia. Thus, vigorous physical activity may reduce the risk for dementia independently of other risk factors. The preventive character of physical activity for neurodegenerative disorders such as Parkinson's disease is not clear yet. While Chen et al. (2005) [12] and Thacker et al. (2008) [13] reported a lower risk to fall sick with Parkinson's disease, Logroscino et al. (2006) [14] did not find an effect of exercise on the risk of PD. However, there is growing evidence that patients suffering from PD benefit from exercise therapy [15].

An active life style also influences poor habits such as smoking, alcohol consumption, and fat nutrition.

Physical activity is defined as any bodily movement produced by skeletal muscles that result in energy expenditure and encompasses both leisure time activity (sports, exercise) [16] and activities of daily life [17]. The WHO has published guidelines for elderly in order to improve cardio-respiratory and muscular fitness [18], bone and functional health, depression, and cognitive decline. According to the WHO, physical activity includes leisure time physical activity, transportation (e.g., walking or cycling), occupational (if the individual is still engaged in work), household chores, play, games, sports, or planned exercise, in the context of daily, family, and community activities. Older adults should be engaged at least in 150 minutes aerobic physical activity of moderate intensity or 75 min of vigorous physical activity throughout the week. To obtain additional health benefits, older adults should increase their moderate intensity of aerobic physical activity to 300 minutes per week, or engage in 150 minutes of vigorous intensity aerobic physical activity per week, or an equivalent combination of moderate and vigorous intensity activity. It has been shown that 20–30 min moderate intensity physical activity on most days result in better physical functions in older adults [19].

The minority of healthy elderly people meet these criteria for activity and even fewer older adults with concomitant diseases exercise regularly with sufficient intensity and frequency [20]. 

Although there are studies showing an increase in leisure time physical activity among elderly in Spain [5], the increase of physical activity did not lead to an increased physical fitness. 

It is still an ongoing debate whether a certain intensity of exercises is necessary to obtain a morbidity lowering effect for cardiovascular and cerebrovascular accidents. While some researchers claim that low intensity exercise such as regular walking, biking, or gardening have a preventive effect [21], other study results indicate that the preventive effect of exercising depends on the intensity of the exercise [22] and an increase of physical fitness. 

According to Lee the additional use of on average 3000–35000 kcal lowers mortality significantly [23]. Some authors found a graded dose response of the volume of physical activity with all-cause mortality, stroke, and several coronary heart disease factors [24]. A clear dose-response between the exercise volume as measured by MET-min × week (−1) and VO_2_max and between the intensity of physical activity and the VO_2_max response has been shown.

However, the energy expenditure of leisure time physical activity did not correlate with the risk of decline in perceived health [21].

Exercise therapy has now been widely accepted as useful tool in the prevention and treatment of several diseases, thus this special issue on aging, physical activity, and disease prevention will now appear annually. Comparable to the first special issue on aging, physical activity and age prevention the current issue covers a wide range of topics. Ten papers have been accepted for publication. 

One paper investigates the role of exercise therapy in the prevention of decline in aging muscle function and in the prevention of glucocorticoid myopathy and muscle unloading. The paper reviews the effects of muscle wasting and the possibilities of exercise therapy. The authors explore the potential molecular effects of exercise therapy on muscle protein metabolism. They postulate that strength exercise can change the renewal of contractile proteins in accordance with the needs of muscle contractile apparatus and endurance exercise might restore the oxidative capacity by stimulation of mitochondrial biogenesis. 

Thus, both, strength and endurance exercise seem to be promising tools for aging-related disease prevention. 

Another paper assesses the correlation between physical activity and frailty phenotypes in females with Parkinson's disease. The authors found that nonfrail patients recorded more physical activity than frail, self-reported physical activity was greater in PD patients than in non-PD subjects. However, physical activity was related to frailty in non-PD subjects only. In PD patients, frailty was rather related to disease associated factors; hence, disease management might be more important. 

The impact of positive and negative social control, support, and perceived strain on physical activity is discussed in another paper. Hierarchical regression analyses revealed that perceived support and perceived strain were not correlated with physical activity. However, age and sex interacted with social control, such that more positive social control was associated with more frequent physical activity for younger men, while more positive and negative social control were significantly associated with less frequent physical activity for older men. There was no association between social control and physical activity among women. The authors concluded that health professionals and social partners should be discouraged from using negative social control because these strategies may be ineffective for women and younger men and may be counter productive for older men. Positive social control strategies might be appropriate for young men and alternative strategies have to be pursued for women.

The authors of another paper provide an overview of “*Convivência*” groups in Brasil. The paper reports on the results of a survey conducted in Florianópolis. Social groups can be crucial for health and well-being in old age. A variety of programmes for older adults supported by the Brazilian National Public Policy in 2003 and established since 2002 were designed to enhance social activities among the older adult population. Community-based social groups known as “convivência” groups were extremely popular. Participating in “convivência” groups helped elderly to be socially engaged and to live actively. 

The purpose of one of the papers was to quantify the extent to which physical activity differed between Veterans and non-Veterans and to determine how diabetes and age influenced this association. After adjusting for age, sex, race and ethnicity, household income, education level, body mass index (BMI), and recent health checkup, Veteran status was associated with a small but significantly larger amount of average weekly moderate physical activity. Diabetes and prediabetes were associated with significantly lower mean levels of both moderate and vigorous intensity physical activity as was increasing age. Veteran status had no impact on the association between diabetes, age, and physical activity.

There is a paper that provides a review on the increase of life expectancy by physical exercise. The authors performed a systematic PubMed search on life expectancy in physically active and inactive subjects, in addition articles comparing life expectancy of athletes compared to that of nonathletes were reviewed. Physical activity reduces many major mortality risk factors including arterial hypertension, diabetes mellitus type 2, dyslipidemia, coronary heart disease, stroke, and cancer. All-cause mortality is decreased by about 30 to 35% in physically active as compared to inactive subjects. The studies suggest that regular physical activity is associated with an increase of life expectancy by 0.4 to 6.9 years. Aerobic endurance athletes showed a greater life expectancy, but it remains unclear if high-intensity sports activities further increase life expectancy. 

The meaning of aging and the development of osteoarthritis is highlighted in another paper. Osteoarthritis (OA) is a major health burden leading to progressive pain and reduced mobility with age as the most prominent risk factor for the development and progression of OA. Inflammatory cytokines play a role in the development of osteoarthritis. Joint movement has been shown to exhibit anti-inflammatory mechanisms. Therefore, physical activity or physiotherapy in the elderly might reduce inflammatory processes and increase muscle mass. 

The surgical treatment of end-stage osteoarthritis in elderly patients was the focus of one of the papers. The authors emphasized that elderly patients may benefit more by total ankle replacement (TAR) than by the alternative ankle arthrodesis, since rehabilitation after TAR is easier than that after ankle arthrodesis. Immobilisation and protection time is shorter and articular and muscle function less affected. Total ankle replacement might be an option to regain mobility and quality of life in elderly patients. 

There is a paper that explores the role of barriers as limiting factor to participation in physical activity in Canadian seniors. The identification of barriers to physical activity and exercise has been used for many decades to explain exercise behaviour in older adults. Typically health concerns are the number one barrier to participation. In contrast to earlier results the current research did not identify a health condition limitation, illness, or injury as a barrier to participation in physical activity. Barriers are not the limiting factor and physical activity programming has to be focused on the health needs of our aging population. 

Another paper reports on the efficacy of a multimodal cognitive rehabilitation programme including psychomotor and endurance training in Parkinson's disease. Executive dysfunction and dementia are mayor problems in Parkinson's disease and more disabling than motor disturbances. The aim of the study was to compare three different cognitive training programmes. The results showed that the multimodal cognitive rehabilitation programme which included physical exercises has been more successful than the other cognitive training programmes. In addition some translation of the improvements into real life has been obtained. 

*Iris Reuter* *Iris Reuter*
